# The association between farming activities, precipitation, and the risk of acute gastrointestinal illness in rural municipalities of Quebec, Canada: a cross-sectional study

**DOI:** 10.1186/1471-2458-10-48

**Published:** 2010-01-30

**Authors:** Yossi Febriani, Patrick Levallois, Suzanne Gingras, Pierre Gosselin, Shannon E Majowicz, Manon D Fleury

**Affiliations:** 1 Unité de recherche en santé publique, Centre Hospitalier Universitaire du Québec, Quebec City, Quebec, Canada; 2 Institut national de santé publique du Québec, Quebec City, Quebec, Canada; 3 Ouranos, Montreal, Quebec, Canada; 4 Centre for Foodborne, Environmental, and Zoonotic Infectious Diseases, Public Health Agency of Canada, Guelph, Ontario, Canada; 5 Department of Population Medicine, University of Guelph, Guelph, Ontario, Canada

## Abstract

**Background:**

Increasing livestock density and animal manure spreading, along with climate factors such as heavy rainfall, may increase the risk of acute gastrointestinal illness (AGI). In this study we evaluated the association between farming activities, precipitation and AGI.

**Methods:**

A cross-sectional telephone survey of randomly selected residents (n = 7006) of 54 rural municipalities in Quebec, Canada, was conducted between April 2007 and April 2008. AGI symptoms and several risk factors were investigated using a phone questionnaire. We calculated the monthly prevalence of AGI, and used multivariate logistic regression, adjusting for several demographic and risk factors, to evaluate the associations between AGI and both intensive farming activities and cumulative weekly precipitation. Cumulative precipitation over each week, from the first to sixth week prior to the onset of AGI, was analyzed to account for both the delayed effect of precipitation on AGI, and the incubation period of causal pathogens. Cumulative precipitation was treated as a four-category variable: high (≥90^th ^percentile), moderate (50^th ^to <90^th ^percentile), low (10^th ^to <50^th ^percentile), and very low (<10^th ^percentile) precipitation.

**Results:**

The overall monthly prevalence of AGI was 5.6% (95% CI 5.0%-6.1%), peaking in winter and spring, and in children 0-4 years old. Living in a territory with intensive farming was negatively associated with AGI: adjusted odds ratio (OR) = 0.70 (95% CI 0.51-0.96). Compared to low precipitation periods, high precipitation periods in the fall (September, October, November) increased the risk of AGI three weeks later (OR = 2.20; 95% CI 1.09-4.44) while very low precipitation periods in the summer (June, July, August) increased the risk of AGI four weeks later (OR = 2.19; 95% CI 1.02-4.71). Further analysis supports the role of water source on the risk of AGI.

**Conclusions:**

AGI poses a significant burden in Quebec rural municipalities with a peak in winter. Intensive farming activities were found to be negatively associated with AGI. However, high and very low precipitation levels were positively associated with the occurrence of AGI, especially during summer and fall. Thus, preventive public health actions during such climate events may be warranted.

## Background

Acute gastrointestinal illness (AGI) is a significant health problem in Canada. Three previous studies conducted in selected residents of Ontario and British Columbia estimated that the monthly prevalence of AGI in the study population ranged between 8 and 10% [1-3]. Moreover, as estimated from selected residents of Hamilton (Ontario), each AGI case brings estimated costs of around $1089 CAD annually [4].

Animal manure contains AGI-causing microbial pathogens. Direct and indirect contact with farm animals or their excreta, such as working or living on a farm, are considered to increase the risk of AGI [5,6]. Furthermore, climate factors such as extreme precipitation have also been associated with AGI [7-9].

In Quebec, according to the *Ministère du Développement durable, de l'Environnement et des Parcs *(MDDEP), seven Quebec watersheds are considered to have high intensive farming activities (dominated by hogs) and be overloaded with manure [10,11], based on the needs in fertilizer of soils in the basin. An increased risk of hospitalization for AGI in the territory with high farming activities has been observed recently, especially in young children, but the ecological study design precluded causal inference [12]. Therefore, this study was conducted to evaluate the association between farming activities, precipitation, and AGI at the individual level. Furthermore, we assessed the effect modification of age, water source, and season on the association between AGI and both farming activities and precipitation. Additionally, we assessed the effect modification of farming activities on the association between AGI and precipitation.

## Methods

### Study design

A cross-sectional telephone survey of randomly selected residents in 54 selected rural municipalities in Quebec, Canada was conducted between April 24^th^, 2007 and April 21^st^, 2008, with an interruption between December 19^th^, 2007 and January 2^nd^, 2008 due to Christmas holidays.

Interviews were conducted by trained interviewers from a Quebec survey firm. Residential telephone numbers with a Quebec area code were randomly sampled. At the beginning of the interview, the number (x) and age of persons living in the household were obtained and a number was assigned to each person by age from 1 (the youngest) to x (the oldest). A person was then randomly selected using this number (e.g. if the number selected was 1, then the youngest person in the house was asked to participate, if the number selected was 2, the second youngest person in the household was asked to participate). This age-based selection procedure was chosen in order to more accurately represent the study population. Proxy respondents were used when the selected participant was (a) <14 years, (b) 14 to 17 years, at the discretion of the parent or guardian, or (c) >17 years, when a developmental or intellectual disability prevented the respondents from answering themselves. Twenty attempts were made to contact the selected individual. Questionaires were administered in French or English, as requested by respondent, using computer-assisted telephone interviewing (CATI) software. The following persons were excluded: (a) children three months old or younger; (b) individuals who lived in institutions, for example chronic care facilities or nursing homes; and (c) individuals who did not speak French or English.

A sample size of 3,500 in each study population (explained below as high intensity farming area or low intensity farming area) for a total of 7,000 people was calculated to estimate a monthly prevalence of AGI of 10%, with an allowable error of 1% and a type 1 error of 5%. Interviews were conducted equally over the 12 month period, with an approximately equal number of interviews completed each day. The average time of interview was about 10 minutes, lasting longer for participants having had an episode of AGI.

Ethical approval for this study was obtained from *Le Comité d'éthique de la recherche de l'Université Laval *(Quebec, Canada).

### Study population

The study population was classified into two groups. The first group consisted of all residents of 31 rural municipalities (total population = 55,434) with high intensive animal farming activities, located in seven watersheds considered by the MDDEP to be overloaded with manure [10]. These watersheds involve the following Quebec rivers: Chaudière, Etchemin, Boyer, L'Assomption, Bayonne, Yamaska, and Nicolet, all located along the St. Lawrence river. A municipality with high intensive animal farming activities is defined as one with: 1) ≥ 25% of its surface area used for farming activities (cultivation and animal breeding); 2) a phosphorus index of ≥ 20 kg/ha per year, and; 3) an animal density of ≥ 2 animal units/hectare (AU/ha) of cultivated land.

The second group consisted of all residents of 23 rural municipalities (total population = 39,413) with low intensive animal farming activities located outside the seven watersheds, in the southern Quebec. A municipality with low intensive animal farming is defined as one with: 1) ≥ 25% of its surface area used for farming activities (cultivation and animal breeding); 2) a phosphorous index ≤ -20 kg/ha per year and; 3) an animal density ≤ 0.4 AU/ha.

The phosphorous index for each municipality was obtained from MDDEP. The phosphorus index is an indicator used to assess the overproduction of manure in a municipality by using the soil-surface phosphorous balance. The index (kg/ha per year) was calculated for each study municipality as the quantity of phosphorus produced by farm animals (kg/year) minus the estimated quantity of phosphorous consumed by cultivated plants (kg/year), divided by the total cultivated area (ha). A positive result indicates a surplus of manure while a negative result indicates a deficit [10].

To focus the study on small municipalities with vulnerable water sources, and to reduce the possibility of exposure contamination between municipalities of the two groups, municipalities were excluded from the study population if they: 1) had a population >5,000; 2) had the St. Lawrence river as their drinking water source as it is a very large river with high diluting power considered to be less contaminated, or; 3) were low intensive farming areas located within about 30 km from the seven watersheds.

### Case definition

A case of AGI was defined as an individual who reported either (a) three or more loose stools in 24 hours, and/or (b) any vomiting, in the 28 days prior to interview. Individuals were excluded from the case group if they had one of the following conditions, which they believed was the cause of their symptoms: (a) a pre-existing illness, previously diagnosed by a physician, in which vomiting or diarrhea are major symptoms; (b) taking a prescription drug or medical treatment known to cause vomiting or diarrhea, or which has vomiting or diarrhea as a side effect; (c) intestinal or stomach surgery; or (d) taking alcohol or other substances, or (e) pregnancy. Individuals excluded from the case group were retained in the non-case group.

### Data collection, sources and variables

The cross sectional telephone survey collected data on symptoms, demographic determinants (age, sex, education, family income, etc), and risk factors of AGI. Individual-level risk factors measured by the questionaire were: water source (community waterworks, domestic wells or others), ingestion of possibly contaminated water, consumption of risky foods (raw meat, fish, or egg; unpasteurized milk or milk cheese), and occupation or living environment in which the individual may have been in contact with human or animal stools.

Municipality-level risk factors were determined as follows. Data on animal farming activities for each municipality, last updated in 2006, were obtained from the MDDEP and the *Ministère de l'Agriculture, des Pêcheries, et de l'Alimentation du Québec *(MAPAQ).

Community water sources (surface or ground water) and type of treatments (filtration, disinfection or both) were obtained from the MDDEP for each study municipality. When respondent's answered 'community waterworks' as their water source, we clarified whether it was community surface or ground water. We also created a variable combining the water source and type of treatments. However, since we did not observed significant variation of risk of AGI across the variable's levels, we conducted the analysis using the water source information only. Water source was thus classified as a four-category variable: community ground water, community surface water, domestic wells, and others.

Daily precipitation (mm) data were obtained from MDDEP's meteorology station surveillance data. The latitude and longitude coordinates of each municipality and station were identified in order to measure the distance between a station and a municipality. Data were extracted from 28 selected stations and assigned to a study municipality from the closest station. Data for the municipalities with high intensive farming activities were acquired from 16 stations while those for municipalities with low intensive farming were acquired from 12 stations. Therefore, some municipalities within the same intensive farming activity group shared station data.

A variable named 'season' was created, based on the date of onset of AGI (for cases) or the date of interview (for non-cases). 'Season' was classified as: winter (December 1^st ^to February 29^th^), spring (March 1^st ^to May 31^st^), summer (June 1^st ^to August 31^st^) and fall (September 1^st ^to November 30^th^) as already used in other studies [7,13].

### Statistical analysis

Individuals who responded 'don't know/not sure' or refused to answer a question were excluded from the analysis for that question. We compared the demographic characteristics of respondents in high versus low intensive farming communities using a Chi-square test. Analyses were weighted by age and sex to the population of the study area according to the data from the 2006 Canadian census of population (Statistics Canada). A *p*-value of ≤ 0.05 was considered statistically significant. All analyses were conducted in SAS version 9.1 [14].

We calculated the monthly prevalence of AGI, defined as the number of AGI cases divided by the total number of respondents, both overall and for each variable category. Healthcare seeking behavior of the AGI cases was also described.

#### Univariate analysis

We assessed the association between each explanatory variable and AGI in a logistic regression model, and odds ratios (OR) were calculated to estimate the risk of AGI. Variables with a *p*-value < 0.2 were selected to be included in the multivariate analysis, except for the variables 'water source' and 'precipitation', which were included in the multivariate regression regardless of the univariate *p*-value. Variables measured at municipality-level were assigned to each respondent within the same municipality. Therefore, for the analysis of the municipality-level variable, a generalized estimating equation (GEE) approach was incorporated into the model and municipality was entered as a repeated effect [15].

#### Multivariate analysis

Multivariate analyses were carried out using logistic regression models, to measure association between AGI and both intensive animal farming activities (high versus low) and cumulative precipitation. The GEE approach was used in all multivariate analyses, and multicollinearity between variables was checked by calculating variation inflation factors and eigenvalues. Effect modification was assessed by using the Wald test for the interaction term included in the model. An effect modification occurs when the effect of an exposure, compared with a reference unexposed condition, depends on the level of one or more other factors [16].

##### Association between intensive animal farming activities and AGI

Multivariate regression was used to assess the association between intensive animal farming activities and AGI. Variables with a univariate *p*-value < 0.2 (except the variable "precipitation") were all included in the initial model. We then deleted the potential confounders one by one starting with the variable with the largest *p*-value. Variables which were statistically significant (*p *≤ 0.05), or considered to be important risk factors of AGI, or which showed confounding effects were kept in the final model. A variable was considered to be confounding if the difference of the OR before and after the variable was entered to the model was ≥ 10%. We then assessed the effect modification of the following variables on the association: age, water source, and season; by entering the interaction term of the variable "farming" and the potentially modifying variable in the final model. Each potentially modifying variable was assessed separately.

##### Association between cumulative weekly precipitation and AGI

Multivariate regression was used to assess the association between cumulative weekly precipitation and AGI. The variable "precipitation" was added to the same final model as that used to assess the association between animal farming activity and AGI. Cumulative weekly precipitation was calculated in six one-week-periods [1-7, 8-14, 15-21, 22-28, 29-35, 36-42 days] prior to the onset AGI (for cases) or the day of interview (for non-cases). These one-week-periods were selected considering (a) the incubation period of the pathogens, which can be up to 4 weeks, and (b) the time it takes for water or food to become contaminated and be ingested [8,13,17]. An analysis using each of the six time periods was used to account for the lagged effect of precipitation on AGI. Each time period was assessed separately and the OR was calculated. Precipitation was also explored as a four-category variable: high (≥90^th ^percentile), moderate (50^th ^to <90^th ^percentile), low (10^th ^to <50^th ^percentile), and very low (<10^th ^percentile) precipitation with low precipitation being the reference category. The season of winter was excluded from the analysis because during the winters in Quebec, precipitation is predominantly in the form of snow rather than rain. The following variables were considered as potentially modifying the association between precipitation and AGI: "farming activity", "age", "water source", and "season". The effect modification of each of those variables on the association was assessed for each time period separately. Furthermore, based on the results, triple interactions between variables (with significant two way interactions) were assessed.

## Results

### Response rate

Of the 15,859 individuals in the sampling frame, 15,519 could be contacted, 12,089 (77.9%) were eligible to participate, and a total of 7,006 participated in the full study. Of the 340 individuals unable to be contacted, we assumed that 77.9% (n = 265) would have been eligible to participate in the study. Thus, the estimate response rate for this study was 56.7% (7,006/[12,089 + 265]).

### Demographic characteristics and the prevalence of AGI

The comparison of demographic characteristics of respondents in each of the rural municipalities with high and low intensive farming is shown in Table 1. Respondents in municipalities with high intensive farming activities were less affluent (*p *< 0.001), less educated (*p *< 0.001) and more culturally homogeneous (*p *= 0.005) than those in municipalities with low intensive farming activities.

**Table 1:** Comparison of demographic characteristics of respondents living in Quebec rural municipalities with high and low intensive animal farming, Canada, April 2007 to April 2008

Demographic characteristic	n	High intensive farming (%)	n	Low intensive farming (%)	n	Total (%)	p*
							
Total	3506	50.0	3500	50.0	7006	100	
							
Sex (n = 7006)							0.625
Male	1573	44.9	1550	44.3	3123	44.6	
Female	1933	55.1	1950	55.7	3883	55.4	
							
Age (years) (n = 7006)							0.134
0-4	171	4.9	130	3.7	301	4.3	
5-9	147	4.2	139	4.0	286	4.1	
10-19	344	9.8	373	10.7	717	10.2	
20-29	312	8.9	296	8.5	608	8.7	
30-39	339	9.7	379	10.8	718	10.2	
40-49	596	17.0	558	15.9	1154	16.5	
50-59	687	19.6	693	19.8	1380	19.7	
60-69	534	15.2	573	16.4	1107	15.8	
70-79	265	7.6	265	7.6	530	7.6	
≥80	111	3.2	94	2.7	205	2.9	
							
Total household income (n = 6185)							< 0.001
<$20000	470	15.0	398	13.0	868	14.0	
≥$20000-<$40000	972	31.0	808	26.5	1780	28.8	
≥$40000-<$60000	816	26.1	784	25.7	1600	25.9	
≥$60000-<$80000	460	14.7	494	16.2	954	15.4	
≥$80000	413	13.2	570	18.7	983	15.9	
							
Education† (n = 6655)							< 0.001
No high school diploma	1153	40.8	884	31.3	2037	36.0	
High school diploma	1035	36.6	1068	37.8	2103	37.2	
College diploma	341	12.1	406	14.3	747	13.2	
University graduate or higher	296	10.5	471	16.6	767	13.6	
							
Cultural group (n = 6910)							0.005
Caucasian	3444	99.6	3419	99.0	6863	99.3	
Others	14	0.4	33	1.0	47	0.7	
							
No. individuals‡ in household (n = 7006)							0.305
1	719	20.5	692	19.8	1411	20.1	
2	1234	35.2	1295	37.0	2529	36.1	
3	557	15.9	526	15.0	1083	15.5	
4	618	17.6	642	18.3	1260	18.0	
≥5	378	10.8	345	9.9	723	10.3	

*Global *p*-value for each demographic characteristic for the total respondents.

†Distribution of survey respondents for each education level was calculated for those aged > 19 years old.

‡Older than 3 months old.

The following gastro-enteric symptoms within 28 days prior to the interview were reported by respondents: vomiting only (n = 90), three or more loose stools in 24 hours only (n = 257), vomiting and/or three or more loose stools in 24 hours (n = 454; corresponded to cases definition). Of the 454 cases, 75 had other conditions and/or pre-existing illness in which diarrhoea and/or vomiting were possibly the symptoms and therefore these cases were excluded from the case group, resulting in a total of 379 cases.

The overall AGI prevalence was 5.59%. The prevalence of AGI in high intensive farming municipalities was 4.71% and in low intensive farming municipalities 6.84% (Table 2). The peak prevalence was observed in children 0-4 years old (Table 2) and in winter and spring (Table 3).

**Table 2:** Prevalence and odds ratios (OR) from the univariate analysis of demographic-related risk factors of acute gastrointestinal illness in Quebec rural municipalities, Canada, April 2007 to April 2008

	W.n*	Prevalence (%)	OR (95% CI)	*p*
				
Overall prevalence	7006	5.59	-	-
				
Sex				
Male†	3576	5.48	1.00	-
Female	3430	5.71	1.04 (0.85-1.28)	0.680
				
Age (years)				<0.001‡
0-4	366	14.18	3.44 (2.31-5.13)	<0.001
5-9	411	10.28	2.38 (1.57-3.62)	<0.001
10-19	1022	5.12	1.12 (0.76-1.66)	0.557
20-29	813	7.33	1.64 (1.13-2.40)	0.010
30-39	867	6.39	1.42 (0.97-2.08)	0.074
40-49†	1194	4.59	1.00	-
50-59	1050	3.86	0.83 (0.55-1.26)	0.392
60-69	701	3.07	0.66 (0.40-1.09)	0.107
70-79	381	2.41	0.51 (0.25-1.04)	0.065
≥ 80	199	2.14	0.45 (0.17-1.23)	0.121
				
Total household income				0.012‡
<$20000†	612	4.56	1.00	-
≥$20000-<$40000	1658	4.65	1.02 (0.66-1.59)	0.925
≥$40000-<$60000	1691	6.68	1.50 (0.98-2.29)	0.062
≥$60000-<$80000	1080	4.97	1.09 (0.68-1.75)	0.704
≥$80000	1157	7.07	1.59 (1.03-2.48)	0.038
				
Education§				<0.001‡
No high school diploma†	1722	3.10	1.00	-
High school diploma	2027	5.08	1.67 (1.19-2.34)	0.003
College diploma	749	5.89	1.95 (1.30-2.94)	0.001
University graduate or	669	6.60	2.20 (1.46-3.32)	<0.001
higher				
				
Cultural group				
Caucasian	6866	5.60	0.86 (0.26-2.79)	0.796
Others†	46	6.48	1.00	-
				
Number of individuals¶ in household				<0.001‡
1†	596	4.82	1.00	-
2	1932	4.07	0.88 (0.56-1.38)	0.582
3	1232	6.46	1.38 (0.88-2.17)	0.162
4	1849	7.30	1.60 (1.04-2.46)	0.031
≥ 5	1398	6.36	1.39 (0.88-2.17)	0.150
				
Length of stay in the municipality				<0.001‡
Less than a year	214	6.86	1.79 (1.03-3.10)	0.039
1 to 5 years	1440	8.60	2.28 (1.80-2.90)	<0.001
6 to 10 years	1005	8.08	2.13 (1.62-2.80)	<0.001
Over 10 years†	4346	3.96	1.00	-
				
Geographic location				
High intensive farming	4100	4.71	0.68 (0.51-0.88)	0.004
Low intensive farming†	2906	6.84	1.00	-

* Weighted n, weighted by age and sex to the population of the study area, according to the data from the 2006 Canadian census of population.

† Reference.

‡ Global *p*-value.

§Prevalence of each education level was calculated for respondents aged > 19 years old.

¶Older than 3 months old.

Health professionals were visited by 14.8% (n = 49) of cases. A physician was visited by 11.6% (n = 44) of cases, <1% of cases (n = 1) visited a nurse, and 1% of cases (n = 4) visited a pharmacist. Twelve cases (3.2%) reported that a stool sample had been taken and seven cases (1.8%) were hospitalized. Fourteen cases took prescribed medications including anti-diarrhoeal (n = 5), anti-nausea (n = 1), antibiotics (n = 5), and other medications such as antacid or anti-inflammatory (n = 3).

### Univariate analysis

Higher income (*p *= 0.012), higher level of education (*p *< 0.001), and higher number of individuals over 3 months old in the household (*p *< 0.001) increased the odds of AGI. The odds of AGI were lower in the older age group and in individuals who had lived for a longer period in the municipality (*p *< 0.001) (Table 2).

Swimming in a public swimming pool or whirlpool was positively associated with AGI (*p *= 0.006). For the climate factors, the odds of AGI in winter and spring were higher compared to fall (*p *= 0.012). Cumulative weekly precipitation of the six lagged one-week-periods was not found to be associated with AGI. Furthermore, water source was not associated with AGI (*p *= 0.143) (Table 3).

**Table 3:** Prevalence and odds ratios (OR) from the univariate analysis of water and climate-related risk factors of acute gastrointestinal illness in Quebec rural municipalities, Canada, April 2007 to April 2008

	W.n*	Prevalence (%)	OR (95% CI)	*p*
				
**Tap water consumption (1 serving = 250 ml)**				0.999‡
No water†	2002	5.46	1.00	-
Less than 4 servings	2231	5.45	1.00 (0.76-1.30)	0.987
Between 4 and 8 servings	2118	5.38	0.98 (0.75-1.29)	0.912
More than 8 servings	255	5.54	1.01 (0.57-1.79)	0.958
				
**Home water treatment**				0.243‡
Use a water treatment device†	1739	5.33	1.00	-
Boil water	286	7.80	1.50 (0.93-2.42)	0.098
None of them	4969	5.56	1.04 (0.82-1.33)	0.724
				
**Water source**				0.143‡
Community ground water†	1980	5.57	1.00	-
Community surface water	1230	6.93	1.26 (0.94-1.69)	0.116
Domestic wells	3532	5.25	0.94 (0.74-1.20)	0.612
Others	238	4.56	0.81 (0.43-1.53)	0.518
				
**Septic systems**				
Municipal sewer†	2995	5.66	1.00	-
Septic tanks	3736	5.56	0.98 (0.79-1.21)	0.851
				
**Swim**				
In a public swimming pool or whirlpool				
Yes	632	8.01	1.54 (1.13-2.09)	0.006
No†	6372	5.35	1.00	-
				
In a lake or a river				
Yes	411	5.48	0.98 (0.63-1.51)	0.921
No†	6593	5.60	1.00	-
				
**Precipitation§**				
Week 1 (days 1-7)				0.824‡
High	663	5.19	0.94(0.60-1.47)	0.790
Moderate	2234	5.26	0.94 (0.72-1.23)	0.672
Low†	1917	5.62	1.00	-
Very low	598	4.63	0.80(0.50-1.27)	0.342
				
Week 2 (days 8-14)				0.962‡
High	616	4.84	0.90 (0.55-1.45)	0.654
Moderate	2114	5.30	0.99 (0.70-1.42)	0.979
Low†	2014	5.43	1.00	-
Very low	644	5.58	1.03(0.67-1.59)	0.894
				
Week 3 (days 15-21)				0.204‡
High	648	5.71	1.05 (0.70-1.57)	0.800
Moderate	2115	5.53	1.02 (0.76-1.37)	0.894
Low†	2209	5.54	1.00	-
Very low	626	3.47	0.61 (0.37-1.00)	0.051
				
Week 4 (days 22-28)				0.822‡
High	579	5.40	1.06 (0.65-1.73)	0.817
Moderate	2151	5.14	1.01(0.75-1.36)	0.958
Low†	2052	5.20	1.00	-
Very low	638	6.26	1.23 (0.80-1.89)	0.835
				
Week 5 (days 29-35)				0.406‡
High	662	6.09	1.04 (0.65-1.68)	0.855
Moderate	2318	4.82	0.82 (0.57-1.18)	0.283
Low†	1831	5.94	1.00	-
Very low	612	4.13	0.69 (0.39-1.20)	0.185
				
Week 6 (days 36-42)				0.323‡
High	603	5.15	0.71 (0.43-1.19)	0.201
Moderate	2116	5.77	1.16 (0.84-1.60)	0.367
Low†	2095	3.59	1.00	-
Very low	609	5.09	0.99 (0.60-1.64)	0.973
				
**Season**				0.012‡
Winter	1546	6.63	1.53 (1.13-2.07)	0.005
Spring	1822	6.40	1.47 (1.10-1.98)	0.009
Summer	1830	5.06	1.14 (0.85-1.96)	0.373
Fall†	1807	4.43	1.00	-

* Weighted n, weighted by age and sex to the population of the study area, according to the data from the 2006 Canadian census of population.

† Reference.

‡ Global *p*-value.

**§ **Each one-week-period [prior to the onset of AGI (for cases) or the interview date (for non-cases)] was modelled separately. Winter season was excluded for the analysis because of very cold winters in Quebec, with precipitation taking mostly the form of snow during that season.

Living in an area of high intensive farming activity was negatively associated with AGI (*p *= 0.004). Working as a day care provider or working in the health sector where one could have been in contact with human stools was positively associated with AGI (*p *= 0.023; Table 4).

**Table 4:** Prevalence and odds ratios (OR) from the univariate analysis of farm-related and other risk factors of acute gastrointestinal illness in Quebec rural municipalities, Canada, April 2007 to April 2008

Variable	W.n*	Prevalence (%)	OR (95% CI)	*p*
				
**Contact with animal or manure**				
Living in farming municipalities				
High intensive farming	4100	4.71	0.68 (0.51-0.88)	0.004
Low intensive farming†	2906	6.84	1.00	-
				
The presence of manure at 300 m away or less from the house				
Yes	2127	6.13	1.17 (0.94-1.45)	0.168
No†	4749	5.31	1.00	-
				
Keeping farm animals				
Yes	965	5.43	0.96 (0.71-1.30)	0.811
No†	6041	5.62	1.00	-
				
Having pets at home				
Yes	4171	5.89	1.15 (0.93-1.41)	0.194
No†	2832	5.16	1.00	-
				
**Consumption of risky food**				
Raw or rare meat				
Yes	438	5.04	0.90 (0.58-1.39)	0.626
No†	6551	5.59	1.00	-
				
Unpasteurized milk or raw milk cheese				
Yes	929	4.16	0.70 (0.50-0.99)	0.042
No†	6076	5.81	1.00	-
				
Raw egg				
Yes	391	7.17	1.33 (0.89-1.98)	0.159
No†	6601	5.49	1.00	-
				
Raw fish				
Yes	533	6.12	1.11 (0.77-1.61)	0.571
No†	6467	5.54	1.00	-
				
**Work**				
As a day care provider or in the health sector where one could have been in contact with human stools				
Yes	337	8.39	1.59 (1.06-2.37)	0.023
No†	6668	5.45	1.00	-
				
In a pet shop, a zoo, an animal clinic, a farm, a slaughterhouse				
Yes	773	5.04	0.88 (0.63-1.24)	0.474
No†	6231	5.66	1.00	-
				
As a chef or assistant chef				
Yes	206	7.41	1.37 (0.80-2.33)	0.250
No†	6799	5.54	1.00	-
				
Take care or baby sit some children				
Yes	1262	5.07	0.88 (0.67-1.16)	0.374
No†	5739	5.70	1.00	-
				
**Travel History**‡				
Yes	372	6.28	1.14 (0.74-1.75)	0.553
No†	6634	5.55	1.00	-

* Weighted n, weighted by age and sex to the population of the study area, according to the data from the 2006 Canadian census of population.

† Reference.

‡ Travelling outside Canada within four weeks prior to the onset of AGI (for cases) or interview date (non-cases)

### Multivariate analysis

The initial multivariate regression model included the following variables: intensive farming activity, the presence of manure 300 meters or less from the house, having pets at home, age, income, education, number of individuals (older than 3 months old) in the household, length of stay in a municipality, season, water source, swimming in a public swimming pool or whirlpool, consumption of unpasteurized milk or raw milk cheese, consumption of raw eggs and working as a day care educator or in the health sector where one could have been in contact with human stools. No multicollinearity between the variables was detected.

#### Association between intensive animal farming activities and AGI

In the final model, we observed that high intensive farming activity was negatively associated with AGI (OR = 0.70, 95% Confidence Interval (CI): 0.51-0.96; Table 5). The negative association was observed particularly in respondents using domestic wells as their primary water source (OR = 0.49, 95% CI: 0.32-0.74). Respondents using community surface water had the highest risk of AGI, however this was not statistically significant (OR = 1.51, 95% CI: 0.97-2.36; Table 6). Age and season were not found to modify the association between farming activity intensity and AGI (*p-*value for the interaction term of: 'age*farming' = 0.930; 'season*farming'= 0.466).

**Table 5:** Adjusted odds ratios (OR) from the multivariate model of the association between intensive animal farming activity and acute gastrointestinal illness in Quebec rural municipalities, Canada, April 2007 to April 2008 (weighted n = 6162)

Variable*	OR	95% CI	*p*
**Farming**			
Low intensive†	1.00	-	-
High intensive	0.70	0.51-0.96	0.027
			
**Water source**			0.265‡
Community ground water†	1.00	-	-
Community surface water	1.24	0.82-1.87	0.298
Domestic wells	0.94	0.70-1.28	0.707
Others	0.85	0.44-1.62	0.621
			
**Age (years)**			<0.001‡
0-4	5.17	2.70-9.87	<0.001
5-9	2.72	1.37-5.41	0.004
10-19	1.54	0.77-3.08	0.225
20-29	1.79	1.04-3.09	0.037
30-39	1.35	0.76-2.42	0.309
40-49†	1.00	-	-
50-59	0.98	0.63-1.56	0.968
60-69	0.72	0.42-1.23	0.230
70-79	0.51	0.26-1.00	0.050
≥80	0.60	0.21-1.69	0.331
			
**Length of stay in the municipality**			0.061‡
Less than a year	1.00	0.52-1.91	0.992
1 to 5 years	1.16	0.80-1.68	0.423
6 to 10 years	1.60	1.12-2.28	0.010
Over 10 years†	1.00	-	-
			
**Season**			0.056‡
Winter	1.52	0.94-2.47	0.088
Spring	1.51	1.00-2.28	0.049
Summer	1.11	0.74-1.67	0.613
Fall†	1.00	-	-

* All variables were introduced in one model and adjusted for family income, education level, consumption of raw eggs, and occupation where one may have been in contact with human stools.

† Reference group.

‡ Global *p*-value

**Table 6:** Modification effect of water source on association between acute gastrointestinal illness and intensive farming activities (*p *for interaction term<0.001) in Quebec rural municipalities (weighted n = 6147), Canada, April 2007-April 2008

Municipality water source	OR*	95% CI for OR	*p*
Community ground water	0.78	0.48-1.25	0.295
Community surface water	1.51	0.97-2.36	0.067
Domestic wells	0.49	0.32-0.74	<0.001
Others	1.02	0.30-3.51	0.962

*Odds ratio for high versus low intensive farming activities adjusted for other risk factors.

#### Association between cumulative weekly precipitation and AGI

We observed significant effect modification of farming activity intensity (*p *= 0.005) and season (*p *= 0.026) on the association between cumulative precipitation of week 4 (days 22-28 before the onset of AGI) and AGI. The effect modification of water source on the association was only borderline significant (*p *= 0.056). The effect modification of age on the association between cumulative precipitation and AGI could not be assessed because the model did not statistically converge.

The cumulative precipitation of week 4 showed a greater risk of AGI when very low precipitation periods occurred in summer (OR = 2.19, 95% CI: 1.02-4.71) compared to low precipitation periods. The risk of AGI was also higher when high precipitation periods occurred in fall season, however the effect was not statistically significant (OR = 2.03, 95% CI: 0.95-4.34). We explored the ORs of AGI for cumulative precipitation according to season for each of the one-week-periods to assess trends. We observed similar and significant effects when high precipitation periods occurred in week 3 (days 15-21 prior to the onset of AGI) of the fall season (OR = 2.20, 95% CI: 1.09-4.44; Table 7).

**Table 7:** Modification effect of season on association between acute gastrointestinal illness (AGI) and cumulative precipitation three and four weeks prior to the onset of AGI in Quebec rural municipalities, Canada, April 2007-April 2008

Week*	W.n†	Precipitation	OR‡ (95% CI) *p*-value for OR			*p*§
				
			Spring	Summer	Fall	
3 (days 15-21)	4770	High	0.83 (027-2.53) 0.749	0.71 (0.28-1.78) 0.457	2.20 (1.09-4.44) 0.028	0.162
		Moderate	1.16 (0.65-2.06) 0.618	1.32 (0.72-2.41) 0.361	0.84 (0.43-1.65) 0.614	
		Low¶	1.00	1.00	1.00	
		Very low	0.49 (0.23-1.03) 0.059	0.72 (0.24-2.18) 0.566	0.46 (0.14-1.46) 0.186	
						
4 (days 22-28)	4771	High	0.75 (0.34-1.98) 0.829	0.92 (0.43-1.98) 0.829	2.03 (0.95-4.34) 0.068	0.026
		Moderate	1.32 (0.77-2.27) 0.305	1.37 (0.70-2.67) 0.356	0.62 (0.31-1.24) 0.180	
		Low¶	1.00	1.00	1.00	
		Very low	0.94 (0.39-2.28) 0.890	2.19 (1.02-4.71) 0.044	0.72 (0.24-2.14) 0.553	

* Each one-week-period [prior to the onset of AGI (for cases) or the interview date (for non-cases)] was modelled separately.

† Weighted n, weighted by age and sex to the population of the study area, according to the data from the 2006 Canadian census of population.

‡ Odds ratio for the precipitation presented according to season. The odds ratio was adjusted for other risk factors. Winter season was excluded for the analysis because of very cold winters in Quebec, with precipitation taking mostly the form of snow during that season.

§ *p*-value for interaction term between precipitation and season.

¶ Reference

We checked the triple interaction of 'precipitation*season*water source' and 'precipitation*season*farming' for the cumulative precipitation of week 4. A significant statistical interaction between precipitation, season, and water source was observed (*p *= 0.046). For respondents using community ground water as their primary water source, high precipitation in summer increased the risk of AGI four weeks later by 2.5 times (OR = 2.51, 95% CI: 1.12-5.62) compared to low precipitation. For those using community surface water, high precipitation in the fall increased the risk of AGI four weeks later by 10 times (OR = 9.64, 95% CI: 2.42-38.37) compared to low precipitation. For those using domestic wells, dry weather (very low precipitation) in summer increased the risk of AGI four weeks later by 3 times (OR = 3.02, 95% CI: 1.12-8.13) compared low precipitation. We did not observed significant statistical interaction between precipitation, season, and farming activity intensity (*p *= 0.491).

## Discussion

The overall monthly prevalence of AGI in our study population was 5.6%. The population living in municipalities with high intensive farming activities had a lower risk of AGI compared to those living in municipalities with low intensive farming activities. Furthermore, heavy precipitation episodes during fall and very low precipitation episodes during summer were found to be positively associated with AGI, but this depended on water source.

The overall monthly prevalence of AGI reported in our study population is consistent with reports from previous studies in Canada and other countries (British Columbia, Canada, 8.8%; United States, 7.6%; Ireland, 3.4%; Australia, 6.4%) [2,18]. The peak prevalence in children under five years and in winter observed in this study has also been observed in previous Canadian studies [3,18]. This may in part due to viral AGI, which is the leading cause of AGI in children especially in winter [19,20]. The lower prevalence in individuals who had lived longer in a municipality may be related with immunity given by constant low-level exposure from sources of pathogen [21-23].

Studies have associated higher intensive farming activities with higher risk of AGI [6,12,24,25]. An ecological study, which evaluated the association between farming activity and hospitalization rates for AGI in a similar study population as ours, found an increased risk of hospitalizations associated with high intensity farming, especially in children [12]. Surprisingly, in the current community-based study, we observed a negative association. This may be in part related to the severity of the AGI cases. In this study, we captured less severe cases. Of 379 cases, only 7 cases were hospitalized. Therefore, it is possible that the risk of AGI in municipalities with high intensive farming activities can only be observed in more severe cases. However, it is also possible that the ecologic study was actually measuring the higher probability that children with AGI get hospitalized more than adults. In this study, we were likely under sampling children and would not capture those hospitalized when phoned for interview. Consequently, it was less likely to see the association between AGI and farming intensity in children.

Immunity may also explain our findings. Protective immunity resulting from repeated exposure from pathogen sources, such as drinking water, has been considered to be an important factor [22,23,26]. It is possible that the residents of high intensive farming municipalities were frequently exposed to pathogens causing AGI, increasing their immunity. We did observe that respondents who had been living in a municipality for over 10 years had lower risk of AGI than those who had been living less than 10 years (Table 2). However, the result was unclear in multivariate analysis (Table 5). Some uncontrolled confounders such as manure treatment, especially composting, may also affect the results. As already known, composting produces heat which removes moisture and kills pathogens [27]. Other possible explanations could include that environmental regulations may be enforced more vigorously or more easily in high intensity farming regions. Unfortunately, we could not evaluate these alternate explanations here.

An increased risk of AGI related with high and very low precipitation episodes has been observed in our study and has also been found in other studies [7-9]. High precipitation may flush manure into surface or groundwater, releasing large microbial loads and thereby leading to contamination of drinking water sources [28]. A very low precipitation period can lead to a lowering of the water table and thereby opening up water flow channels, allowing groundwater to become contaminated by surface water. Less dilution of sewage effluents and animal waste can also result from a long dry period which may consequently contaminate water sources [7,29].

Studies in Canada and the United States have linked weather with AGI. The study by Thomas et al. [8] observed that the risk of a waterborne AGI outbreak increased (OR= 2.283; 95% CI 1.216 - 4.285) for precipitation events greater than the 93^rd ^percentile. Curriero et al. [9] observed that 51% of waterborne disease outbreaks, which were mostly AGI, were preceded by precipitation events above the 90^th ^percentile (*p *= 0.002). Furthermore, Nichols et al. [7] found a significant association between a waterborne disease outbreak and excess cumulative rainfall in the seven days prior to the outbreak (*p *= 0.001), as well as a significant association between low rainfall and the outbreaks (*p *= 0.002). Our study highlights some possible intervention windows in the summer and fall seasons for preventive actions, after either very dry or very wet episodes.

Our study has several strengths. Compared to the previous ecological study on the association between AGI and farming activity in the similar study population [12], this study included more individual-level data, allowing for the control of more individual-level confounders. The other strength of this study was the administration of the survey in both English and French, thus minimizing information bias due to language problems.

Our study has several limitations. When we evaluated the association between AGI and precipitation, we extrapolated precipitation data from available weather stations. Consequently, exposure data may not be precise given the high spatial variability of precipitation and the low density of such stations in rural areas. Also, water consumption was estimated at the moment of interview and did not necessarily reflect the actual water consumption before the occurrence of AGI symptoms. The ecological and aggregated nature of animal density data may also lead to a misclassification of exposure to microbes from animal source. One may live in a municipality with high animal density without being exposed to microbes by manure spreading, depending on a number of variables such as well protection, topography and drainage. All the preceding factors could over or underestimate exposure, leading to non-differential misclassification. Furthermore, we excluded 75 individuals who presented with other conditions (e.g. pregnancy) or with pre-existing illness from the case group, but kept them as non-cases. This may cause a misclassification bias. If some or all of the excluded cases were actually AGI cases, the prevalence of AGI and association observed will be underestimated. Another limitation was that we did not include individuals who lived in institutions in our survey. Since most of these individuals are elderly, and are thus more vulnerable to AGI, this may also lead to the underestimate prevalence of AGI and associations observed.

## Conclusions

The study demonstrates that AGI represents a potentially significant health burden in Quebec rural municipalities, especially in young children. Higher and lower precipitation patterns in the fall and summer may contribute significantly to the occurrence of AGI in Quebec. However, their contributions may vary according to individual's drinking water source. Therefore, these results might be a stimulus for drinking water providers to increase their precautions, especially after high precipitation events. Domestic well owners should also be more careful in dry spells and consider eventually alternate sources for drinking water. Further investigation is needed to clarify this association, as well as the negative association observed between intensive farming activity and AGI.

## Competing interests

The authors declare that they have no competing interests.

## Authors' contributions

YF performed statistical analysis and drafted the manuscript. PL and PG conceived of the study, and participated in its design and coordination. SG participated in the design of the study and planned data analysis. SM and MF participated in the design of the study. All authors were involved in interpreting the results and revising the manuscript. All authors read and approved the final manuscript.

## Pre-publication history

The pre-publication history for this paper can be accessed here:

http://www.biomedcentral.com/1471-2458/10/48/prepub
